# Superinfection with SARS-CoV-2 Has Deleterious Effects on Mycobacterium bovis BCG Immunity and Promotes Dissemination of Mycobacterium tuberculosis

**DOI:** 10.1128/spectrum.03075-22

**Published:** 2022-10-06

**Authors:** Rachel E. Hildebrand, Shaswath Sekar Chandrasekar, Mariah Riel, Bubacarr J. B. Touray, Sophie A. Aschenbroich, Adel M. Talaat

**Affiliations:** a Department of Pathobiological Sciences, School of Veterinary Medicine, University of Wisconsin—Madison, Madison, Wisconsin, USA; b Pan Genome Systems, Madison, Wisconsin, USA; PHRI-Rutgers

**Keywords:** BCG, *Mycobacterium tuberculosis*, SARS-CoV-2, superinfection, pathogenesis, virulence

## Abstract

An estimated one-third of the world’s population is infected with Mycobacterium tuberculosis, with the majority being vaccinated with Mycobacterium bovis BCG. Severe acute respiratory syndrome coronavirus 2 (SARS-CoV-2) remains a threat, and we must understand how SARS-CoV-2 can modulate both BCG immunity and tuberculosis pathogenesis. Interestingly, neither BCG vaccination nor tuberculosis infection resulted in differences in clinical outcomes associated with SARS-CoV-2 in transgenic mice. Surprisingly, earlier M. tuberculosis infection resulted in lower SARS-CoV-2 viral loads, mediated by the heightened immune microenvironment of the murine lungs, unlike vaccination with BCG, which had no impact. In contrast, M. tuberculosis-infected tissues had increased bacterial loads and decreased histiocytic inflammation in the lungs following SARS-CoV-2 superinfection. SARS-CoV-2 modulated BCG-induced type 17 responses while decreasing type 1 and increasing type 2 cytokines in M. tuberculosis-infected mice. These findings challenge initial findings of BCG’s positive impact on SARS-CoV-2 infection and suggest potential ramifications for M. tuberculosis reactivation upon SARS-CoV-2 superinfection.

**IMPORTANCE** Prior to SARS-CoV-2, M. tuberculosis was the leading infectious disease killer, with an estimated one-third of the world’s population infected and 1.7 million deaths a year. Here, we show that SARS-CoV-2 superinfection caused increased bacterial dissemination in M. tuberculosis-infected mice along with immune and pathological changes. SARS-CoV-2 also impacted the immunity of BCG-vaccinated mice, resulting in decreased interleukin-17 (IL-17) levels, while offering no protective effect against SARS-CoV-2. These results demonstrate that SARS-CoV-2 may have a deleterious effect on the ongoing M. tuberculosis pandemic and potentially limit BCG’s efficacy.

## INTRODUCTION

Tuberculosis (TB), caused by infection with Mycobacterium tuberculosis, is one of the leading causes of death, with an estimated annual mortality rate of ~1.7 million (1). Unfortunately, the tuberculosis pandemic collides with the recent coronavirus disease 2019 (COVID-19) pandemic caused by severe acute respiratory syndrome coronavirus 2 (SARS-CoV-2). The emergence of SARS-CoV-2 in 2019 resulted in 246 million confirmed cases and 5 million deaths by the end of October 2021 (2). Historically, both in the 1918 influenza pandemic and with H1N1 nearly 100 years later, bacterial lung superinfections and coinfections were the leading causes of death during viral pandemics (3, 4). Recently, more clinical data on SARS-CoV-2 coinfections and superinfections have emerged, including bacterial superinfections, which have been reported in ~16% of those who are critically ill (3). As expected, superinfection with another bacterial, viral, or fungal agent increases the likelihood of mechanical ventilation and leads to a higher risk of death for SARS-CoV-2 patients (3). So far, the most common bacterial agents reported in conjunction with SARS-CoV-2 superinfection have been Acinetobacter spp., Pseudomonas, and Escherichia coli (3). A few case reports were reported on SARS-CoV-2 superinfection in tuberculosis patients despite reduced hospital visits and access to medical care as a result of the current pandemic (5, 6). The experiments detailed in this report represent our efforts to understand the immunopathogenesis of M. tuberculosis when a host is superinfected with SARS-CoV-2.

The global burden of M. tuberculosis combined with the global burden of SARS-CoV-2 suggests that superinfections are inevitable and frequent, and the current lack of clinical data is likely due to difficulties inherent to TB diagnosis as well as the impacts of the COVID-19 pandemic on tuberculosis care (7, 8). Historically, only about ~5 to 10% of people develop active tuberculosis during the course of their life; however, it is too early to see if this percentage will remain unchanged due to altered tuberculosis pathogenesis in patients with SARS-CoV-2 superinfections (9). In a global study spanning 16 countries and 33 TB centers, decreases in active and latent tuberculosis cases were noted in the first 4 months of the COVID-19 pandemic due to reduced health care access and services as a result of the pandemic compared to the previous year (7). However, in several cohort studies spanning multiple countries, M. tuberculosis and SARS-CoV-2 dual infections point toward an increased risk of death, a shorter time to death, and a longer time to recovery in patients with a dual diagnosis of tuberculosis and COVID-19, at least in the studied populations (5, 6). Data from an early case study suggested that M. tuberculosis infection also increases susceptibility to SARS-CoV-2 (5). Fortunately, the vaccine currently licensed for the control of tuberculosis, Mycobacterium bovis bacillus Calmette-Guérin (BCG), is the most administered vaccine worldwide, with an estimated global coverage of 90% in 1990 (10). Early during the COVID-19 pandemic, lower disease burdens in countries with high BCG vaccination rates sparked speculation that BCG provided some nonspecific innate trained immunity that was applicable to SARS-CoV-2 (11, 12). However, some later clinical and epidemiological studies suggested no effect on mortality and morbidity (13–16). Although BCG is protective only against childhood tuberculosis, it also shows nonspecific protection against other conditions, including urinary bladder cancer (11, 17), and it could also have an immunomodulatory effect on the progression of COVID-19.

Both M. tuberculosis and SARS-CoV-2 have immunomodulating abilities with the potential to change the outcome of the course of each disease in superinfected hosts. M. tuberculosis can cause T-cell exhaustion as well as uncontrolled high levels of proinflammatory cytokines contributing to debilitating lung damage, key risk factors that may contribute to the susceptibility to and severity of COVID-19 (18–20). On the other hand, SARS-CoV-2 causes immunosuppression and cytokine storms, which could contribute to the pathogenesis of M. tuberculosis (20–22). Additionally, some COVID-19 treatment regimens include immunosuppressive drugs that could trigger tuberculosis reactivation (20, 23). Recently, the progression of TB to a more active form was noted in a patient with COVID-19 (24). In this report, we examined the immunopathogenesis of BCG immunity and tuberculosis progression in hosts superinfected with SARS-CoV-2 using the murine model of tuberculosis. Despite its known immunomodulatory effects, BCG had little effect on SARS-CoV-2 mortality and morbidity. In both the active and chronic stages of murine tuberculosis, the SARS-CoV-2 burden was lower in the lungs and spleen, but it did not have any effect on the mortality and morbidity of SARS-CoV-2 infection. On the other hand, at both of these infection time points, tuberculosis-superinfected mice showed increased bacterial dissemination to extrapulmonary tissues, with lower type 1 and higher type 2 cytokine responses than those of the controls, triggered by SARS-CoV-2 superinfection, in conjunction with decreasing histiocytic inflammation in murine lungs. Altogether, these findings support the urgent need to study SARS-CoV-2 superinfection in M. tuberculosis-infected hosts.

## RESULTS

### BCG vaccination has a limited impact on SARS-CoV-2 pathogenesis.

**FIG 1:**
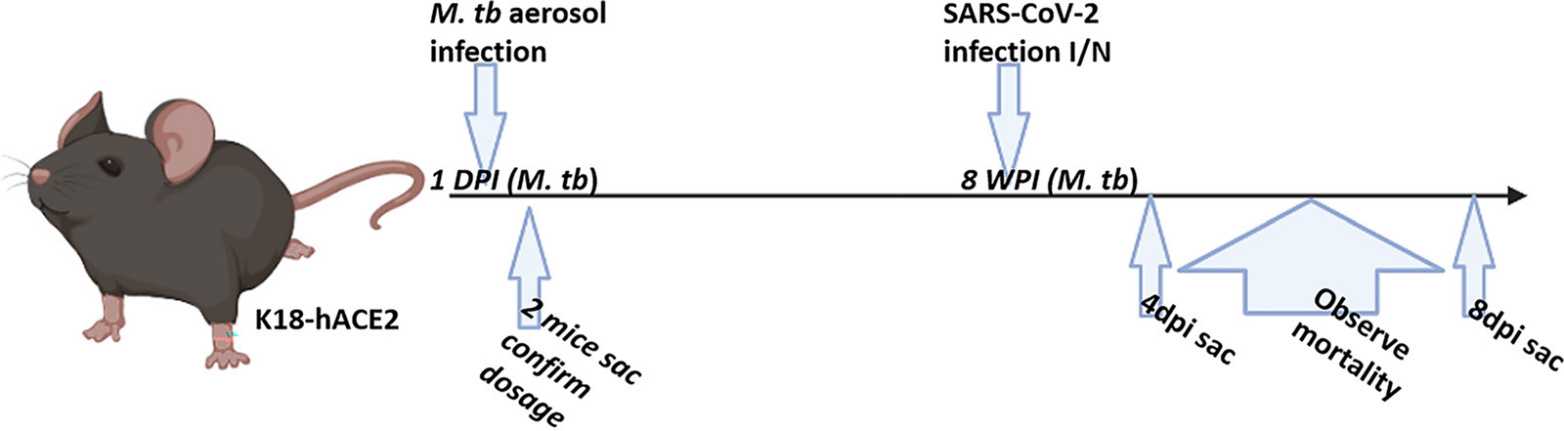
Experimental design of superinfection studies. Mice were infected with 100 CFU/lung via aerosol with M. tuberculosis Erdman (*M. tb*) or vaccinated with 10^6^ CFU BCG via subcutaneous injection. Four and eight weeks after infection (weeks postinfection [wpi]) with M. tuberculosis or vaccination with BCG, mouse groups were challenged with 10^3^ PFU of SARS-CoV-2. Half of the mice were sacrificed at 4 days postinfection (dpi) for sampling, and the remaining mice were sacrificed when they became moribund or at 8 dpi as an endpoint. There were at least 5 mice per group for both the 4-dpi and mortality curves. For the 4-wpi experiment, only the mortality curve was done, and surviving mice were sacrificed at 8 dpi. I/N, intranasally. (Image created with Biorender.com.)

**FIG 2:**
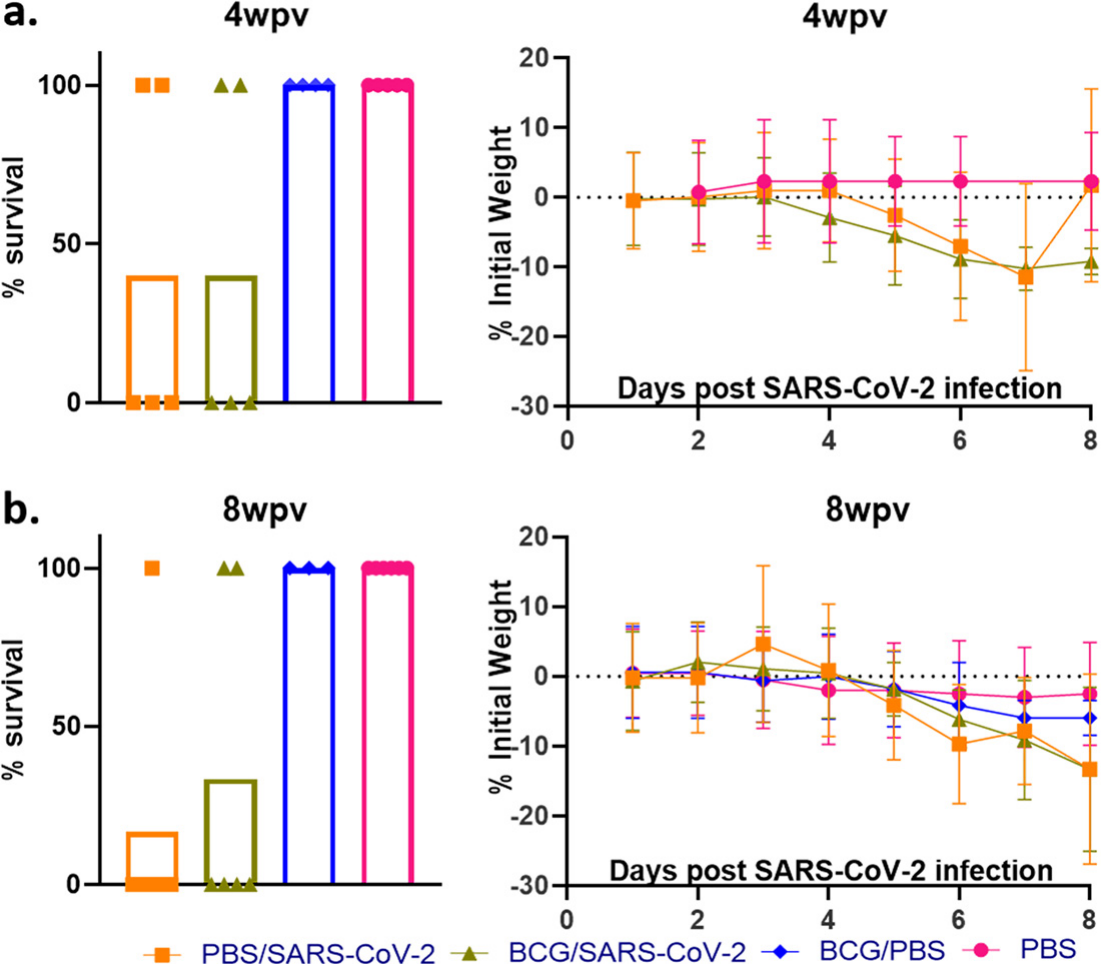
BCG vaccination has a limited impact on SARS-CoV-2 infection. (a) Percent survival at the experimental endpoint (left) and percent change of initial weights (right) for mice challenged with SARS-CoV-2 after 4 weeks postvaccination (wpv). Day 0 refers to the day of SARS-CoV-2 challenge. (b) Similar to panel A but for mice challenged with SARS-CoV-2 8 weeks after vaccination with BCG. All 4-wpi groups contain 5 mice, and 8-wpi groups contain 6 mice, with the exception of the BCG/PBS group, which has 3. The survival curve statistics were calculated using the Kaplan-Meier method, and the weight changes were compared on each day using ANOVA, with standard deviations reported for each time point.

**FIG 3:**
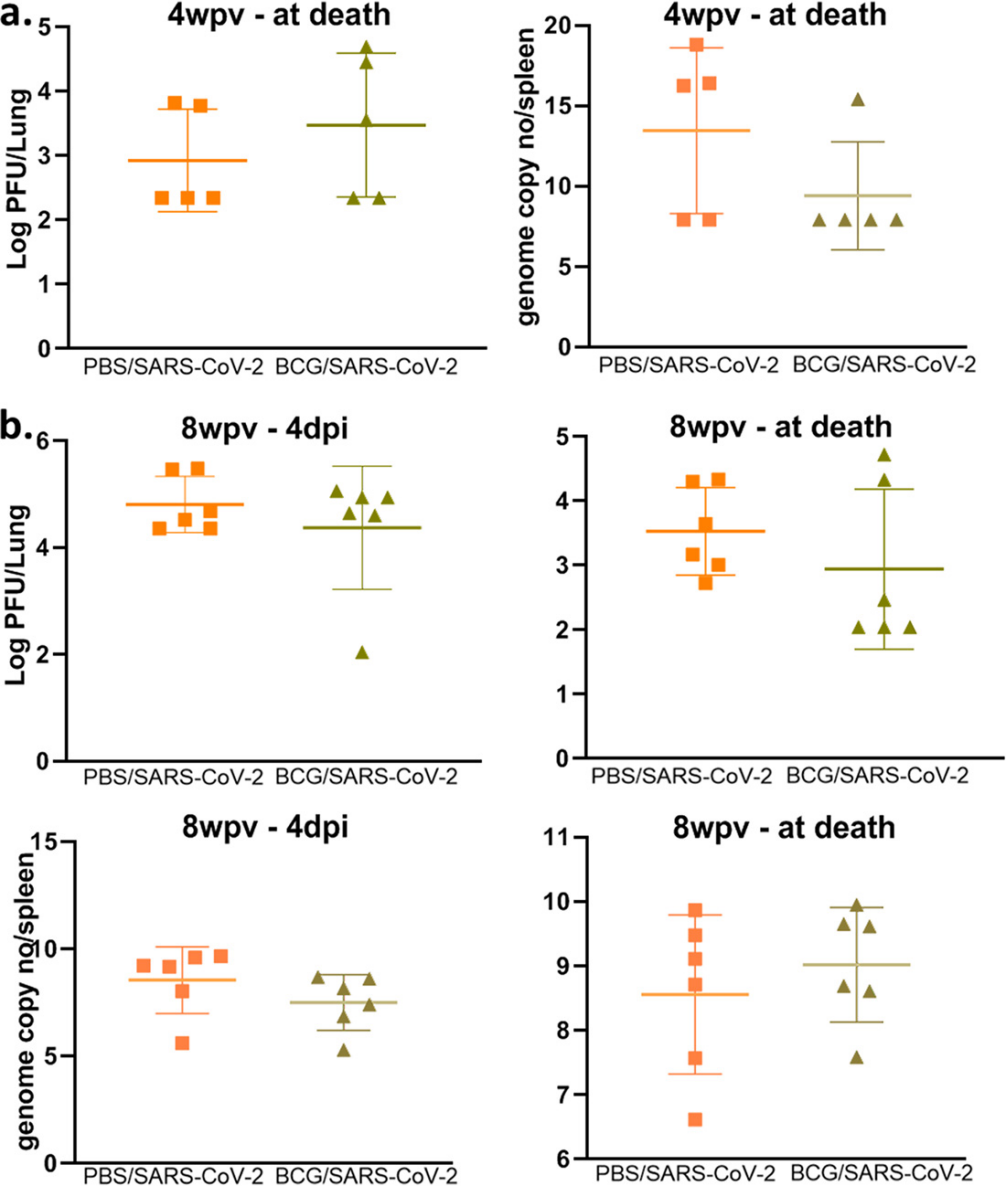
BCG vaccination does not impact SARS-CoV-2 viral loads. (a) Viral loads in murine lungs (PFU per lung) and spleen (genome copy numbers per spleen) from mice sacrificed at the time of death between 5 and 7 days after infection with SARS-CoV-2 and 4 weeks after vaccination with BCG. (b) Viral loads in murine lungs and spleens from mice sacrificed at 4 dpi and at the time of death between 5 and 7 dpi 8 weeks after vaccination with BCG. A log scale was used for all viral loads. *P* values for normally distributed data were determined by ANOVA, while *P* values for nonnormally distributed data were determined by a Kruskal-Wallis test. *P* values of <0.05 (*), <0.01 (**), and <0.0001 (****) were considered significant. See Materials and Methods for further details. Lung viral loads were measured by a plaque assay, while spleen loads were measured by real-time PCR.

**FIG 4:**
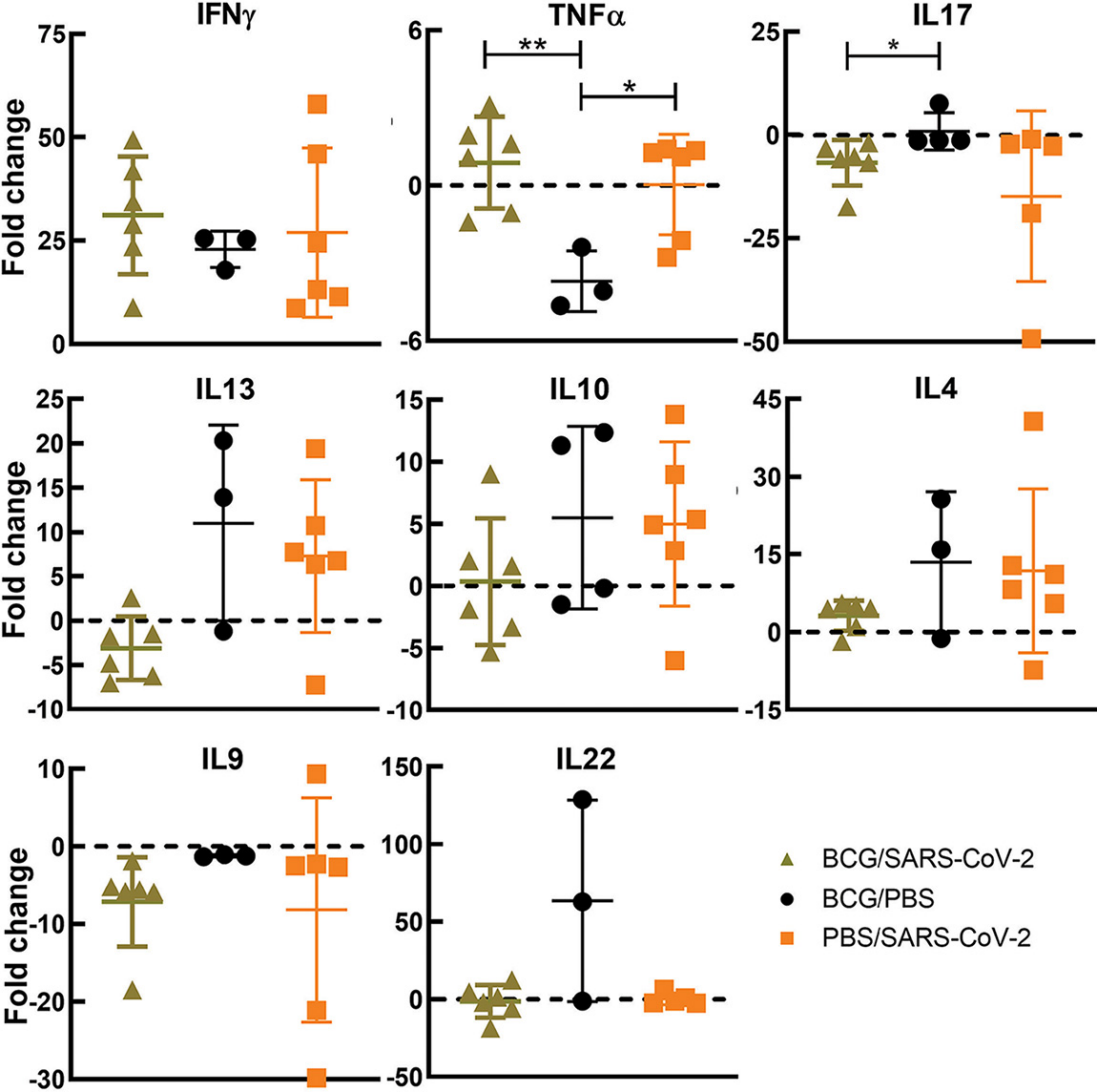
BCG vaccination causes no significant change in SARS-CoV-2 cytokine profiles. Cytokine expression levels at 8 wpv normalized to naive age-matched mice were determined by quantitative real-time PCR (qRT-PCR) for key cytokines involved in the pathogenesis of SARS-CoV-2 and BCG protection. Each panel indicates one cytokine for the BCG-only, BCG/SARS-CoV-2 superinfection, or SARS-CoV-2-alone groups. *P* values for normally distributed data were determined using ANOVA, while *P* values for nonnormally distributed data were determined using a Kruskal-Wallis test. *P* values of <0.05 (*), <0.01 (**), and <0.0001 (****) were considered significant. See Materials and Methods for further details.

To evaluate the efficacy of BCG in preventing SARS-CoV-2 infection and disease, mice were immunized with 10^6^ CFU administered subcutaneously. Mice were subsequently challenged intranasally with SARS-CoV-2 at 4 and 8 weeks postvaccination (wpv) (Fig. 1). Mice from each group were sacrificed at 4 days postinfection (dpi) and also when they became moribund (referred to as “at death” in the figures), at between 5 and 8 dpi. At between 5 and 8 dpi, >50% mortality was observed, with no significant difference between BCG/SARS-CoV-2 and SARS-CoV-2 alone (Fig. 2). Weight loss rates were similar between the groups, although mice in BCG/SARS-CoV-2 group in the 4-wpv study did not recover their weight as quickly as the mice in the SARS-CoV-2 group (Fig. 2). BCG-immunized mice did not have any observed weight loss, as expected. The viral loads of SARS-CoV-2 in the lungs were analyzed at 4 dpi and at death. There was no significant difference between the viral loads in the lungs or the spleen of mice in the BCG/SARS-CoV-2 group and SARS-CoV-2-alone-infected mice (Fig. 3). Furthermore, lung lesions in these groups were typical of those of murine SARS-CoV-2 infection, with a mild, multifocal expansion of alveolar septa by inflammatory cells and scattered type II pneumocyte hyperplasia. There was no discernible difference in lung inflammatory responses between the BCG/SARS-CoV-2 and SARS-CoV-2 groups (see Fig. S1 in the supplemental material). BCG colonization in murine organs (lung, liver, and spleen) was below detectable levels when animals were sacrificed at 4 and 8 wpv. Additionally, there were no histologically apparent lesions in the lungs, livers, or spleens of BCG-vaccinated mice alone, compared to uninfected (phosphate-buffered saline [PBS]-treated) mice. Immunologically, in the BCG/SARS-CoV-2 group, interleukin-9 (IL-9), IL-13, and IL-17 were downregulated and tumor necrosis factor alpha (TNF-α) was upregulated relative to the BCG group at the 8-wpv time point (Fig. 4), although only TNF-α and IL-17 exhibited statistical significance between the BCG/PBS and BCG/SARS-CoV-2 groups.

### Preinfection with M. tuberculosis reduces the SARS-CoV-2 viral load.

**FIG 5:**
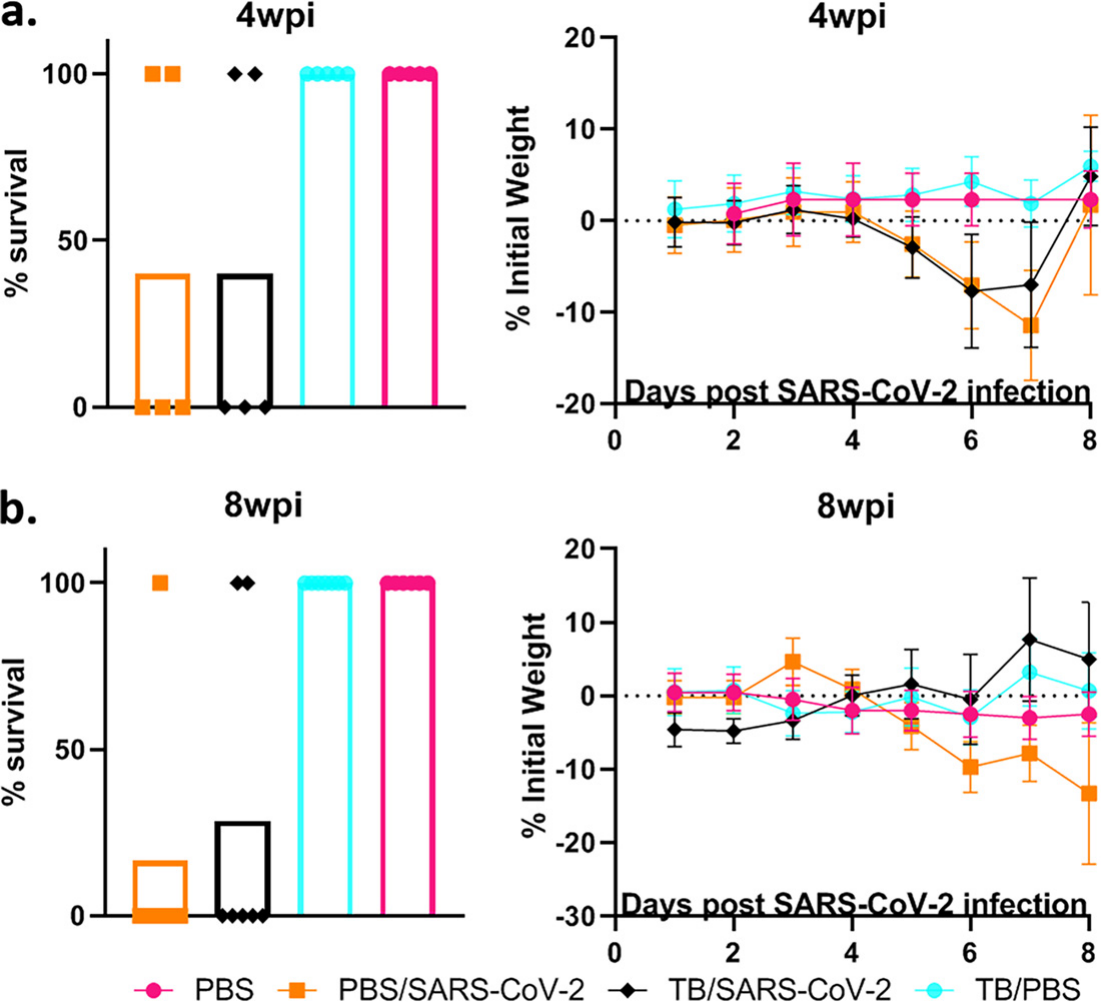
Previous M. tuberculosis infection has a limited impact on SARS-CoV-2 infection outcomes. (a) Percent survival at the experimental endpoint (left) and percent change from initial body weights (right) for mice challenged with SARS-CoV-2 4 weeks after infection with M. tuberculosis. Day 0 refers to the day of SARS-CoV-2 challenge. (b) Percent survival and percent of body weight change, similar to panel A, for mice challenged with SARS-CoV-2 8 weeks after infection with M. tuberculosis. All 4-wpi groups contain 5 mice, and 8-wpi groups contain 6 mice, with the exception of the BCG/PBS group, which has 3. The survival curve statistics were calculated using the Kaplan-Meier method, and weight changes were compared on each day using ANOVA, with standard deviations reported for each time point.

**FIG 6:**
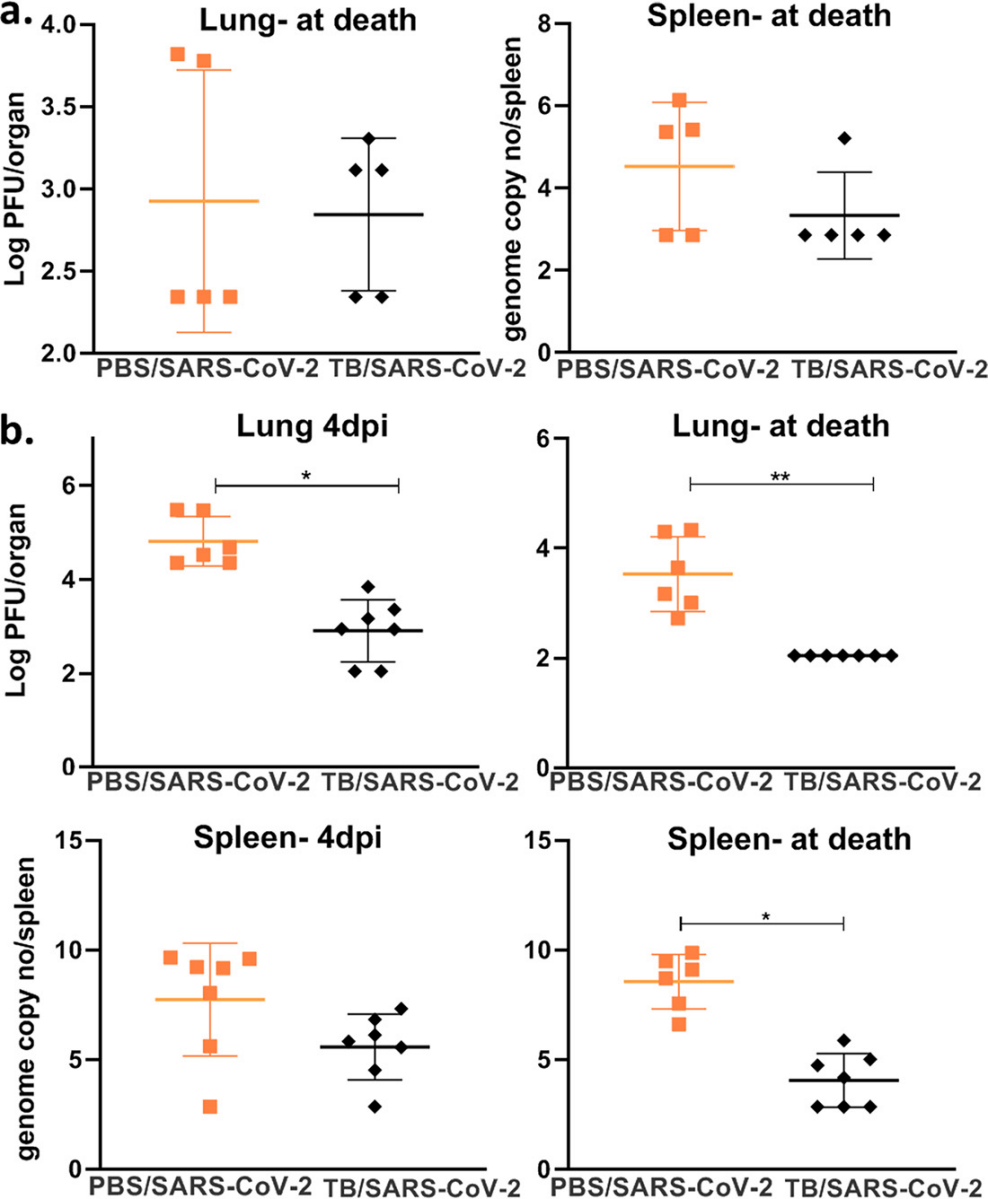
Previous M. tuberculosis infection lowers the SARS-CoV-2 burden. (a) Viral loads in murine lungs (PFU per lung) and spleen (genome copy numbers per spleen) from mice sacrificed at the time of death between 5 and 7 days after infection with SARS-CoV-2 and 4 weeks after infection with M. tuberculosis. (b) Viral loads in murine lungs and spleen from mice sacrificed at 4 dpi and at the time of death between 5 and 7 dpi 8 weeks after infection with M. tuberculosis. A log scale was used for all viral loads. *P* values for normally distributed data were determined using ANOVA, while *P* values for nonnormally distributed data were determined using a Kruskal-Wallis test. *P* values of <0.05 (*), <0.01 (**), and <0.0001 (****) were considered significant. See Materials and Methods for further details. Lung viral loads were measured by a plaque assay, while spleen loads were measured by real-time PCR.

To test if previous M. tuberculosis infection worsened the outcomes of SARS-CoV-2 infection, mice were first infected with a target of 100 CFU/lung of M. tuberculosis Erdman and then superinfected with SARS-CoV-2 at either 4 or 8 weeks postinfection (wpi). No significant differences in mortality or weight gain of mice were seen between the TB/SARS-CoV-2 and SARS-CoV-2 groups, suggesting that previous M. tuberculosis infection has a limited effect on SARS-CoV-2 mortality (Fig. 5). Viral loads were both quantified using plaque-forming assays and confirmed via quantitative real-time PCR (qRT-PCR) in case of potential cytopathic effects (CPEs) caused by M. tuberculosis in the plaque assay. The TB/SARS-CoV-2 group had lower viral loads in both the lung and spleen at 4 wpi, with significant reductions seen at 8 wpi (Fig. 6). As expected, the TB infection group suffered no weight loss or mortality up to 8 wpi.

### SARS-CoV-2 modulates host control of M. tuberculosis.

**FIG 7:**
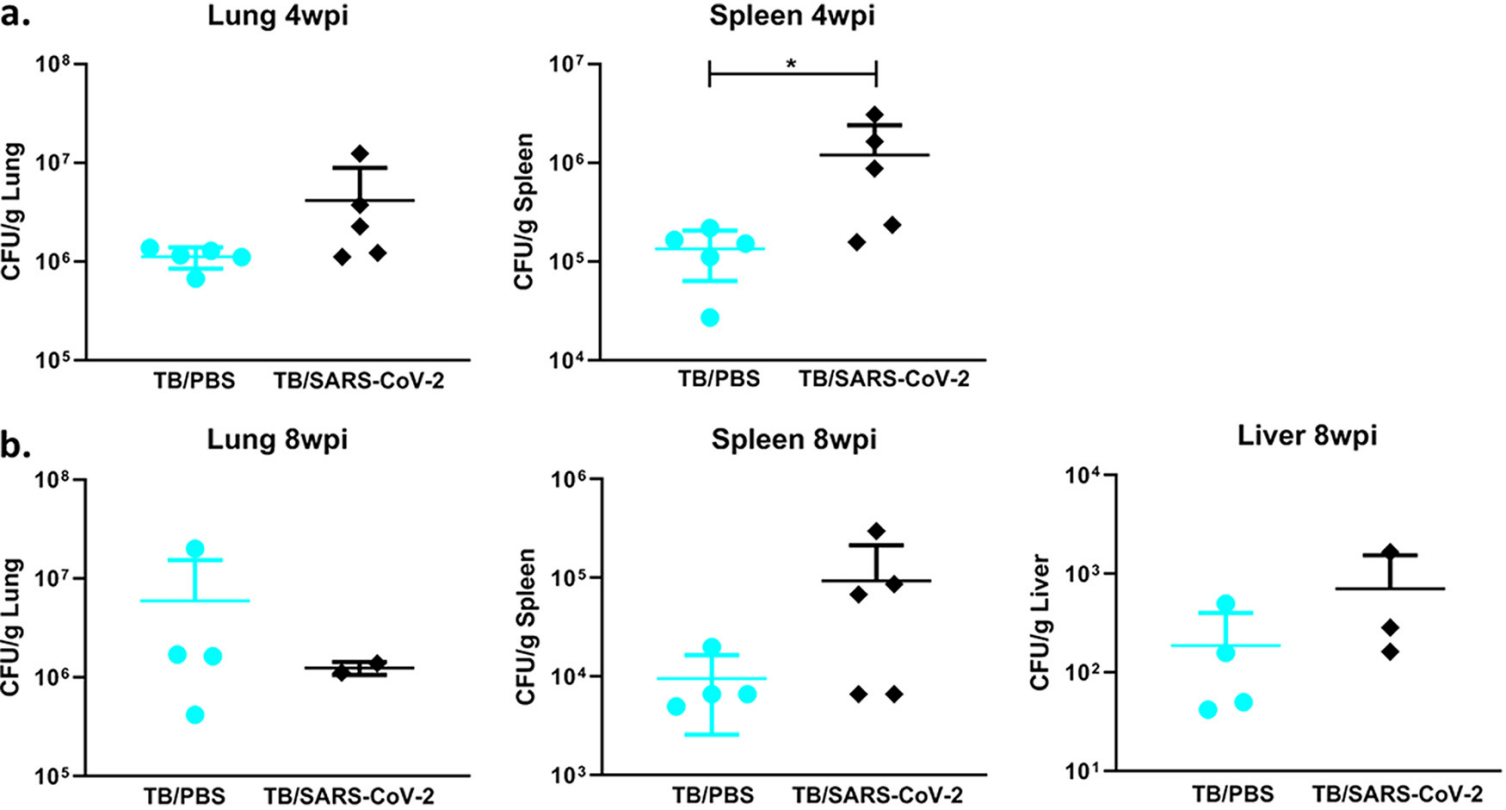
SARS-CoV-2 superinfection results in increased dissemination of M. tuberculosis. (a) Bacterial loads in murine lung and spleen (CFU per organ) from mice sacrificed at the time of death between 5 and 7 days after infection with SARS-CoV-2 4 weeks following M. tuberculosis infection. (b) Bacterial loads in the lung, spleen, and liver from mice sacrificed at the time of death between 5 and 7 days after infection with SARS-CoV-2 8 weeks following M. tuberculosis infection. Statistics were calculated using a Mann-Whitney test, and *P* values of <0.05 (*) were considered significant.

To test whether SARS-CoV-2 infection can modulate bacterial loads and the immune response and host reaction to M. tuberculosis, samples of the lung, spleen, and liver were collected at 4 dpi (days after infection with SARS-CoV-2) and at death for mice at both active (4 wpi) and chronic (8 wpi) TB stages. In the TB/SARS-CoV-2-superinfected groups, there was a trend toward increases in bacterial loads across the liver, lung, and spleen, compared to the TB-alone group, at both active and chronic time points, which did not reach statistical significance, with the exception of the spleen at 4 wpi (Fig. 7). Differences in bacterial loads were especially notable in the spleen, where a full log difference in CFU was observed between the TB/SARS-CoV-2 and TB groups (Fig. 7).

**FIG 8:**
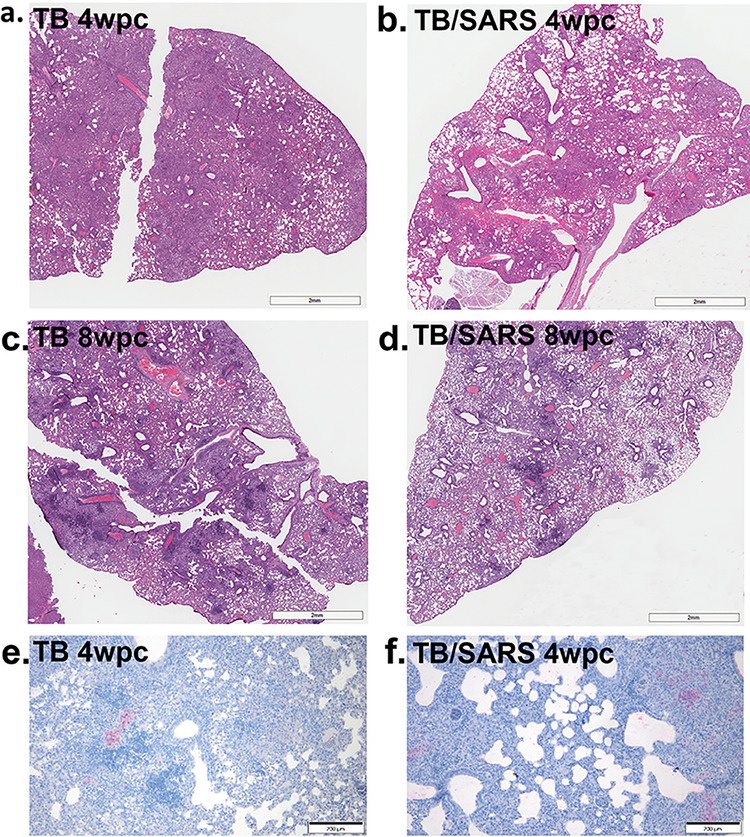
Superinfection results in lesions representative of M. tuberculosis infection but with decreased histiocytic inflammation. Shown are hematoxylin and eosin (H&E)-stained lung sections from M. tuberculosis (TB)- and M. tuberculosis- and SARS-CoV-2 (TB/SARS)-infected mice sacrificed at the time of death between 5 and 7 days after SARS-CoV-2 infection. (a) TB lung at 4 wpi. (b) TB/SARS-CoV-2 lung at 4 wpi. (c) TB lung at 8 wpi. (d) TB/SARS-CoV-2 lung at 8 wpi. (e) Immunohistochemistry (IHC) targeting M. tuberculosis in representative tissue sections of the TB group at 4 wpi. (f) IHC targeting M. tuberculosis in representative TB/SARS-CoV-2 tissue sections at 4 wpi. wpc, weeks postchallenge.

The histologically evident lesions in the lungs of the TB-alone and TB/SARS-CoV-2 groups were consistent with those of a typical M. tuberculosis infection in murine models of infection. Specifically, there were variably well-defined to poorly defined, multifocal to coalescing foci of histiocytic inflammation at 4 wpi, which progressed to include high numbers of foamy macrophages at the chronic stage of infection (8 wpi) (Fig. 8; Fig. S2). For the active stage (4 wpi), the TB/SARS-CoV-2-superinfected group showed less severe pulmonary lesions, with a lower percentage of lung tissue being affected with histiocytic inflammation than in the TB-alone group (Fig. 8; Fig. S2 and Table S1). A similar trend toward decreased pulmonary inflammation was noted in the superinfected group at the chronic stage (8 wpi), which consisted of small, scattered, and discrete histiocytic foci despite high variance in lesion severity within the TB-only infection group (Fig. S2). Interestingly, immunohistochemistry (IHC) used to identify the localization and spread of M. tuberculosis within these pulmonary histiocytic foci revealed a greater dispersion of bacilli within these inflamed regions in the TB/SARS-CoV-2 groups, despite an overall lower percentage of pulmonary tissue affected, than in the TB-alone groups at 4 wpi (Fig. 8). Although superinfection at either time point resulted in a trend toward decreased histiocytic inflammation, superinfection did not appear to have a significant impact on the organization of histiocytic foci, with no significant differences in the location or degree of the accompanying lymphocyte infiltration.

### SARS-CoV-2 shifts the cytokine profile associated with M. tuberculosis.

**FIG 9:**
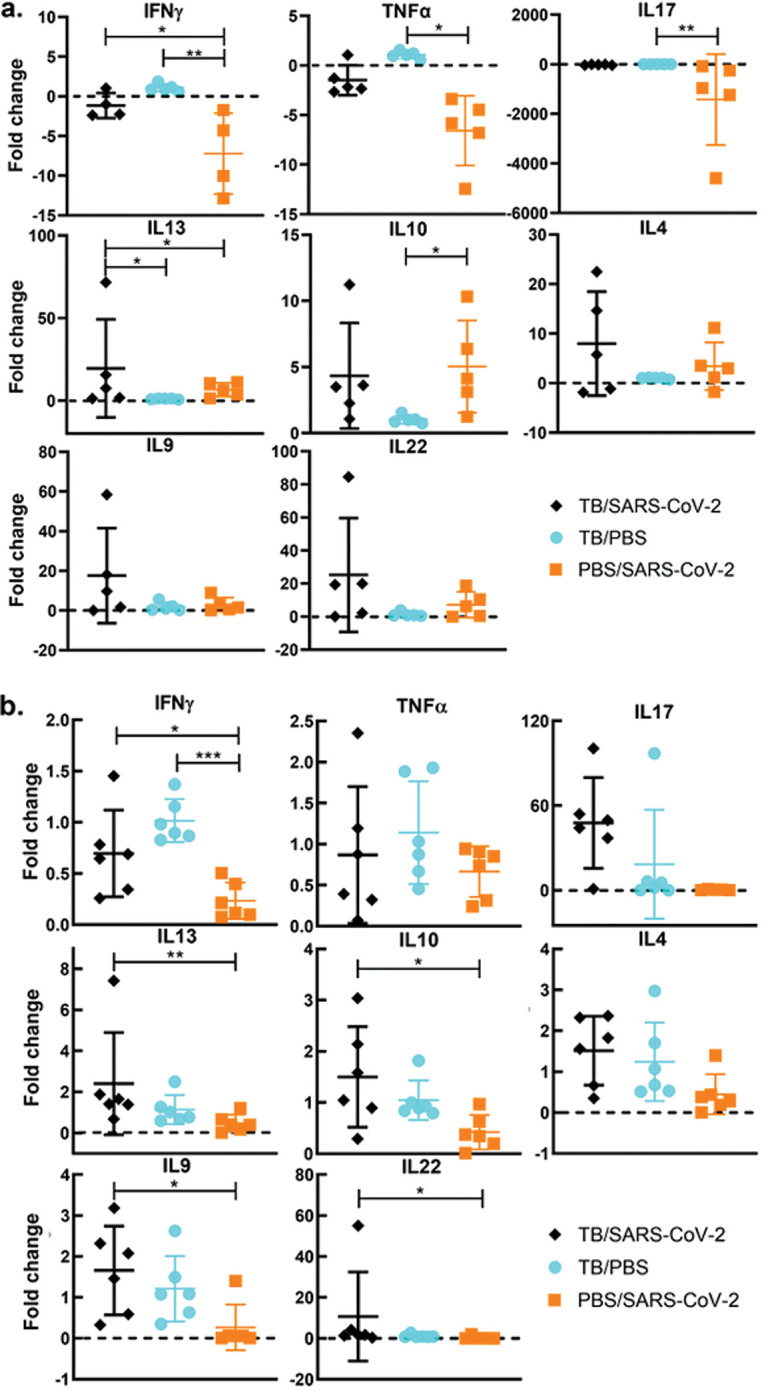
Tuberculosis-superinfected lungs had reduced type 1 responses and increased type 2 responses. (a) Cytokine expression levels 4 weeks after infection with M. tuberculosis following superinfection with SARS-CoV-2 normalized to the levels in the TB-alone group determined by quantitative real-time PCR (qRT-PCR) for key cytokines involved in the pathogenesis of M. tuberculosis- and SARS-CoV-2-infected mice. Each panel indicates one cytokine for the TB-only, TB/SARS-CoV-2, or SARS-CoV-2-alone groups. (b) Cytokine expression levels 8 weeks after infection with M. tuberculosis following superinfection with SARS-CoV-2 normalized to the levels for a naive age-matched mouse determined by qRT-PCR. *P* values for normally distributed data were determined using ANOVA, while *P* values for nonnormally distributed data were determined using a Kruskal-Wallis test. *P* values of <0.05 (*), <0.01 (**), and <0.0001 (****) were considered significant. See Materials and Methods for further details.

Due to SARS-CoV-2 immunomodulatory capabilities, the hypothesis was that superinfection would change the immune microenvironment of M. tuberculosis-infected mice. To test this hypothesis, qRT-PCR was used to determine cytokine levels, which were normalized to those of the TB-alone group average at each examined TB stage. Across the two time points, only one significant difference occurred between the TB/SARS-CoV-2 and TB-alone groups: IL-13 at 4 wpi. However, other trends were consistent across the time points. At the active stage (4 wpi), there were decreases in interferon gamma (IFN-γ) and TNF-α levels in superinfected mice compared to the TB group, although the levels were still higher than those in the SARS-CoV-2-infected mice (Fig. 9a). Cytokines associated with type 2 responses, IL-4 and IL-13, were upregulated in the superinfected group relative to the TB-alone group (Fig. 9a). IL-10 levels were similar between the superinfected and SARS-CoV-2-alone groups but were higher than those in the TB-alone group. IL-17 levels were significantly downregulated in the SARS-CoV-2-alone group and slightly downregulated in the TB/SARS-CoV-2 groups relative to the TB-alone group (Fig. 9a).

At the chronic stage (8 wpi), trends seen at the active stage remained similar throughout the chronic stage, with some exceptions. The IFN-γ levels in TB/SARS-CoV-2-infected mice were reduced compared to those in the TB-alone group but were still higher than those in the SARS-CoV-2-alone group (Fig. 9b). Interestingly, IL-17 did not follow this pattern where the TB/SARS-CoV-2 group had high levels of expression relative to those in all other groups. It also differed from the active stage in that the superinfected group had the highest IL-17 expression levels. For the type 2 cytokines IL-13, IL-4, and IL-10, the TB/SARS-CoV-2 group had increased levels compared to those of the TB-alone group (Fig. 9b). It was observed that SARS-CoV-2 superinfection increased both IL-22 and IL-9 levels relative to those of the TB-alone group at 4 wpi and 8 wpi (Fig. 9b).

## DISCUSSION

Since an estimated one-third of the world’s population is infected with M. tuberculosis, and SARS-CoV-2 has caused over 5 million deaths worldwide in less than 2 years (1), research into how SARS-CoV-2 superinfection will affect the transmission, morbidity, and mortality of M. tuberculosis infections and vaccinations is of paramount importance. Early during the COVID-19 pandemic, there was speculation on BCG vaccination and its nonspecific immunity being protective against SARS-CoV-2, especially as countries with high BCG vaccination rates initially correlated with lower caseloads and numbers of deaths related to COVID-19. A recent study examining BCG protection against SARS-CoV-2 showed no reduction in lung viral loads but reduced weight loss compared to SARS-CoV-2 infection alone (25). In contrast, our findings demonstrate that SARS-CoV-2-specific pulmonary lesions and clinical signs are not impacted by previous BCG vaccination despite the modest modulation of specific cytokines. Our findings are corroborated by another recent paper demonstrating that subcutaneous BCG vaccination has little impact on SARS-CoV-2 challenge after 6 wpv (26). These findings experimentally support the more recent epidemiological observations that BCG vaccination offers no protection against SARS-CoV-2 (13–16). Although there is no protective vaccine against tuberculosis in adults, BCG vaccination protects children against fatal TB infections, with variable protection in adults (10, 27). To our knowledge, no other study has yet examined the SARS-CoV-2-mediated immunomodulation of BCG with respect to IL-17 (25, 26). The decreased levels of IL-17 shown in this study, a cytokine that correlates with BCG-mediated immunity against TB and is a predictor of recovery from tuberculosis, are worrisome for BCG’s future efficacy (27, 28). To better understand these dynamics, studies exploring the longevity of these immune changes and their effects on TB protection should be done. We also examined whether SARS-CoV-2 infection would result in changes in the levels of BCG host colonization. Unsurprisingly, BCG organ colonization levels were below the limit of detection, congruent with data from previous studies (29), another indication of the safety of BCG even if given during the SARS-CoV-2 pandemic. BCG is not normally evaluated in K18-hACE2 mice, and this change could have affected our results. However, the little apparent pathology, the low circulating cytokine levels, and the bacterial loads at or below the limit of detection are consistent with our unpublished work in BCG-vaccinated C57BL/6 mice. Other studies have shown that recombinant BCG vaccination in mice (30), revaccination with BCG in a clinical trial (31), and other routes of vaccination (intravenous) affect SARS-CoV-2 morbidity and mortality (26), demonstrating BCG’s unique off-target effects and potential as an adjuvant or booster. However, our study attempted to best experimentally represent what BCG’s effect would be clinically on SARS-CoV-2 given current vaccination regimens, which are intradermal and given at birth.

Unlike BCG vaccination, previous infection with M. tuberculosis led to reduced SARS-CoV-2 viral loads in the lung, which may be the result of circulating cytokines associated with ongoing M. tuberculosis infection. Specifically, these cytokines may have primed the host’s cell-mediated immune responses to hamper viral replication and dissemination, resulting in a reduced capacity of SARS-CoV-2 to exacerbate existing pulmonary lesions associated with previous M. tuberculosis infection. Coinfection with human rhinovirus demonstrated a similar outcome where the rhinovirus was protective against SARS-CoV-2 replication, which is congruent with the results of another recent study on superinfection with TB/SARS-CoV-2 (32, 33). However, we found that the reduced viral loads in TB/SARS-CoV-2-infected mice did not correlate with differences in weight loss or mortality rates compared to the SARS-CoV-2-only group, unlike coinfection with rhinovirus. The difference in mortality may be specific to the mouse model used (34), a limitation of this study that could be avoided in future studies when alternative models (e.g., hamsters or nonhuman primates) are used.

In this study, the TB/SARS-CoV-2 superinfection group had a unique bacterial burden and histologic features that were different from those of the SARS-CoV-2-only or M. tuberculosis-only groups. In the superinfection groups, there were increases in mycobacterial loads in most tissues that reached significant differences from those of the M. tuberculosis-alone group in the spleen. In conjunction with the increased bacterial loads in pulmonary and extrapulmonary tissues, across both time points, the superinfection group overall demonstrated a trend toward decreased M. tuberculosis-specific histiocytic inflammation in the lung compared to the M. tuberculosis-alone group. Interestingly, while pulmonary inflammation was decreased and more scattered in the TB/SARS-CoV-2 group (particularly at the 8-wpi time point), numerous and dispersed bacilli per lesion were visualized by IHC analysis in this group. Although wild-type mouse models, like the one used in this study, do not form the typical granulomas seen in human tuberculosis, the decreased inflammation and greater bacterial presence within these pulmonary histiocytic foci in the superinfected groups may indicate histiocyte dysregulation or immunomodulation, allowing the increased replication of M. tuberculosis and dissemination to extrapulmonary organs, as seen in the spleen. Other studies examining reactivation and granuloma integrity via TNF-α blockade saw significant changes on a 4-week timeline typical for M. tuberculosis (35). Our study compared lung lesions and bacterial burdens between the time points of 5 and 8 days postinfection and represents a relatively unexamined and short time course for M. tuberculosis disease. Interestingly, a similar study examining superinfection at 4 wpi in K18-hACE2 mice did not show lesion differences or increases in bacterial burdens (32). This may be due to differences in the M. tuberculosis strains used: our use of a clinical isolate instead of the laboratory strain (H37Rv) may represent a more virulent strain that better disseminates within the time frame examined. The short time course and differences between these two studies highlight the importance of studies monitoring recovered superinfected mice where the true impact of the immunological changes and their impact on lesion development and bacterial dissemination can be observed. In our immunological analysis, we show that previous TB infection mediates SARS-CoV-2 impacts on cytokine regulation, with only one significant change occurring between the TB/SARS-CoV-2 and TB-alone groups. However, there were trends shown with cytokine changes alongside other findings in this study (e.g., lower histiocytic inflammation and higher bacterial dissemination) that are associated with TB reactivation and progression. Cytokines associated with type 2 responses, such as IL-13 and IL-10, were upregulated in the superinfected mice. This cytokine profile is usually associated with disseminated M. tuberculosis and progressive infection, similar to that noted previously for active tuberculosis (36, 37). Interestingly, TNF-α and IFN-γ levels were lower in superinfected mice, demonstrating a decrease in type 1 and an increase in type 2 responses overall. In a study by O. Rosas Mejia et al., similar patterns were shown in their immunological analysis of IFN-γ, TNF-α, and IL-10 at the 30-dpi time point (32). IL-17 is the only cytokine that was not consistently affected across the two time points. In general, IL-17 is a proinflammatory cytokine that is needed for the control of and immunity against M. tuberculosis, but high levels are associated with severe tissue damage as well as negative outcomes in SARS-CoV-2 patients (38–40). High levels of IL-17 expression mediated by SARS-CoV-2 infection in a superinfection could induce uncontrolled pulmonary inflammation and trigger cytokine storms (41). In the present study, with our murine model of infection, various levels of IL-17 across the two time points were observed. At 4 wpi, the TB/SARS-CoV-2 group had IL-17 levels that were higher than those in the SARS-CoV-2 group and lower than those in the TB group, while at 8 wpi, the IL-17 levels were downregulated relative to those in the TB-alone group. This difference could be due in part to the cyclical nature of host immune responses to TB infection and the higher circulating levels of IL-17 at the time of SARS-CoV-2 infection (42). Differences in mortality and morbidity between these two time points are not significant, although larger studies may help characterize the role of IL-17 if any. Although the roles of both IL-9 and IL-22 in the pathogenesis of both tuberculosis and SARS-CoV-2 remain unclear (43, 44), both were upregulated in the superinfected mice at both time points, an outcome that warrants future studies.

**FIG 10:**
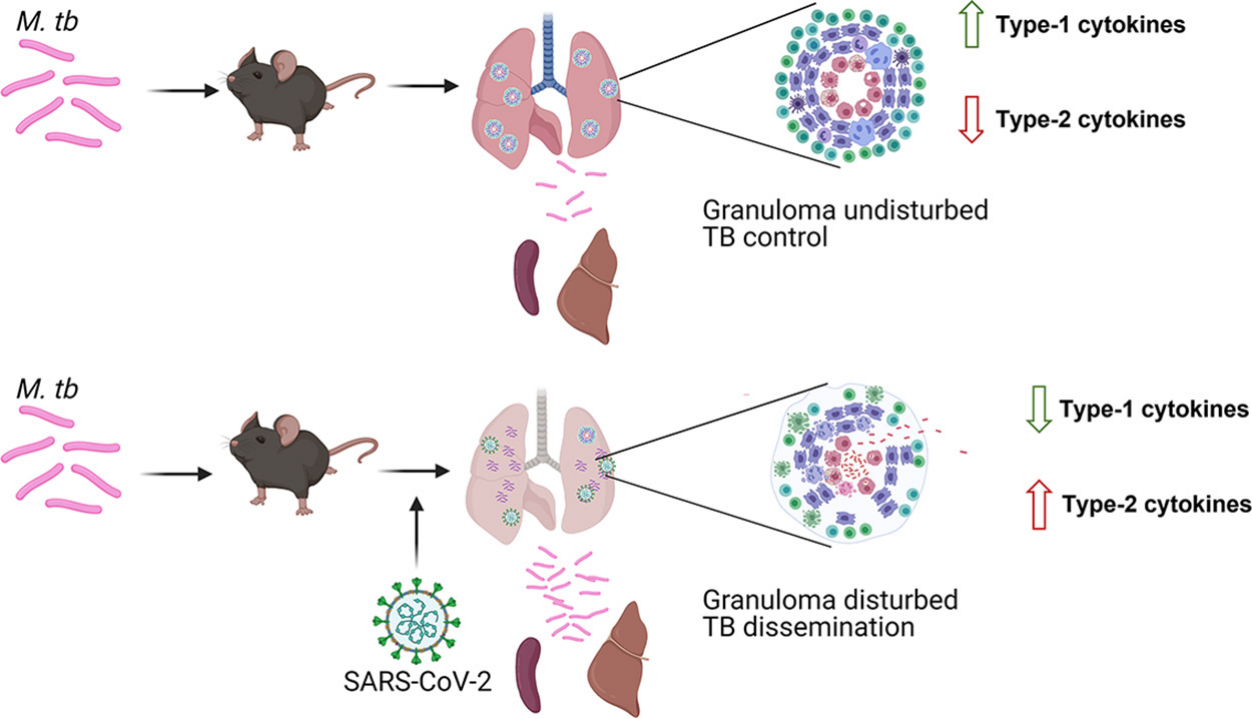
Proposed mechanism of M. tuberculosis dissemination. When infected with M. tuberculosis alone, mice develop granulomas that contain bacilli. These granulomas are maintained by a balance that includes higher type 1 and type 17 levels and lower type 2 and type 9 levels. When mice are infected with SARS-CoV-2, this balance is disturbed, resulting in the downregulation of the type 1 cytokines and the upregulation of type 2 and type 9 cells. This results in granuloma dysregulation and bacterial escape and dissemination. (Image created with Biorender.com.)

Although our immunological analysis is limited to RNA expression levels, the patterns shown are consistent, with the exception of IL-17, across two time points. Our findings supplement our existing hypothesis of SARS-CoV-2 superinfection causing immune dysregulation, potentially resulting in granuloma collapse and the subsequent activation and dissemination of M. tuberculosis (45). A model for such dynamic changes expected in TB/SARS-CoV-2-infected hosts is illustrated in Fig. 10. It remains to be examined if a similar profile is exhibited in humans or when other models of superinfection are used. Previously, a similar paradigm was demonstrated for TB patients who suffered from helminth infections, which exhibited similar cytokine profiles and increased bacterial dissemination (46, 47). Epidemiologically, helminth superinfections are associated with TB reactivation and more severe disease (46, 47). A similar outcome may be observed in the upcoming years with the reactivation of tuberculosis cases caused by SARS-CoV-2 superinfection. In fact, a recent observational study in northern India showed an increase in the number of TB cases despite the lower rate of testing as a result of the COVID-19 pandemic, and a recent case study demonstrated TB reactivation in a patient with latent TB infection (LTBI) after SARS-CoV-2 infection (24, 48). In humans, SARS-CoV-2 infection causes a proinflammatory response that can lead to a cytokine storm in severe disease (22). Likewise, M. tuberculosis also causes a proinflammatory response, although the host response to M. tuberculosis infections has a reduced magnitude and a cyclical pattern (49). Similar to tuberculosis with HIV superinfection in mice (50), we observed increased levels of IL-10 and IL-6 in superinfected tissues compared to those in both the SARS-CoV-2-alone and tuberculosis-alone groups.

Overall, both active and chronic stages of TB infection were examined following SARS-CoV-2 superinfection in mice to model active and latent stages of tuberculosis in humans. Latent infections are the most common form of TB infection as only about 5% to 10% of people who are infected develop an active infection (9). The many underlying causes of TB reactivation point to a dysregulation of the immune system potentially resulting in granuloma collapse and the dissemination of virulent mycobacteria (51). Early on, it was estimated that HIV superinfection in tuberculosis patients could increase the risk of M. tuberculosis reactivation in 50% of the cases (52). Our results demonstrate that SARS-CoV-2 infection coincides with increased bacterial loads in all organs (compared to the TB-only groups), supporting widespread dissemination. Interestingly, this increased bacterial replication in the superinfected groups is concomitant with reduced M. tuberculosis-specific histologic lesions; these findings imply an additional risk factor for tuberculosis patients in the setting of a SARS-CoV-2 pandemic. Unfortunately, BCG vaccination had little to no effect on the course of SARS-CoV-2 pathogenesis. More studies on the host microenvironment level are needed to further elucidate the cellular and genetic changes in tuberculosis hosts following superinfection with SARS-CoV-2.

## MATERIALS AND METHODS

### Cells and viruses.

Vero E6 cells were maintained in Dulbecco’s modified Eagle’s medium (DMEM) supplemented with 10% fetal bovine serum (FBS) and penicillin-streptomycin (D10) at 37°C at 125 rpm with 5% CO_2_ in ventilated plastic flasks. The SARS-CoV-2 isolate USA-WA1/2020 (lineage A), a gift from Jorge Osorio, was propagated in Vero E6 cells. The stock was titrated using the TCID_50_ (50% tissue culture infectious dose) method and also expressed as PFU (described below).

### Bacterial growth conditions.

Cultures of M. tuberculosis Erdman or M. bovis BCG (Pasteur TMC 1011), received from the ATCC, were started from frozen stocks and grown to an optical density at 600 nm (OD_600_) of ~1.0 in Middlebrook 7H9 medium (Remel, Lenexa, KS) with 10% albumin-dextrose-catalase (ADC) and 0.05% Tween 80. Cultures were then washed twice with PBS, frozen in storage medium (10% glycerol, 0.05% Tween 80, and 0.85% NaCl in deionized water), and stored at −80°C. The dosage was determined via plating, and cultures were suspended in PBS for infection/vaccination. Tissues for mycobacterium isolation were homogenized in PBS, plated onto Middlebrook 7H10 medium (Remel, Lenexa, KS) with 10% ADC, and incubated at 37°C for 4 to 6 weeks. Negative plates were left for 8 weeks to confirm sterility. Some tissue samples were cultured on Middlebrook 7H10 medium supplemented with 10 mg/L amphotericin B, an antifungal agent.

### Mouse infections.

The effects of superinfection were evaluated in transgenic C57BL/6 hACE2 mice (6 to 8 weeks old) obtained from the Jackson Laboratory and maintained in biosafety level 2 or level 3 containment for working with BCG or M. tuberculosis, respectively. Mice were either vaccinated with 10^6^ M. bovis BCG Pasteur 1101 bacteria subcutaneously or infected via aerosol using the Glas-Col inhalation exposure system (Glas-Col, LLC, Terre Haute, IN) with a virulent strain, M. tuberculosis Erdman (target dosage of 100 CFU/lung). For the 4-week postinfection/postvaccination time point, there were 5 mice per group. For the 8-week postinfection/postvaccination time point, there were 5 to 7 mice per group per sacrifice time point. M. tuberculosis Erdman infection was confirmed by sacrificing 2 mice at 1 day postinfection, harvesting the lungs, and determining the CFU as described above. At 4 and 8 weeks postvaccination/postinfection, mice were challenged with SARS-CoV-2 intranasally at a dosage of 10^3^ PFU (the 50% lethal dose [LD_50_] in this model). Mice were weighed on the day of SARS-CoV-2 challenge and every day thereafter. Mice were euthanized 4 days after infection with SARS-CoV-2 isolate USA-WA1/2020 or when moribund, as determined by a weight loss of 20% of their body weight or severe lethargy, as done previously for SARS-CoV-2 infections (26, 32). Healthy control mice were sacrificed in conjunction with their superinfected counterparts for consistency. At 8 dpi and 14 dpi for the 4-week and 8-week time points, respectively, the surviving mice were sacrificed. Mice were euthanized via isoflurane overdose and cervical dislocation. The lung, spleen, liver, intestine, and kidney were collected for qRT-PCR, histopathology, and colony counts.

### Quantification of viral loads.

Tissue samples were homogenized to a final 1-mL suspension in serum-free medium (Opti-MEM) with sterile zirconia beads and clarified by low-speed centrifugation at 800 × *g* for 10 min at 4°C, and virus titers were determined in Vero E6 cell monolayers grown in 96-well plates. Vero E6 cells were seeded (25,000 cells/well) into a 96-well plate and incubated overnight at 37°C in a CO_2_ incubator. One hundred microliters of a 10-fold serially diluted tissue suspension was added to each well in a quadruplicate format for 1 h at 37°C and replaced with fresh complete DMEM. The plates were incubated in a CO_2_ incubator at 37°C for 3 days, after which cytopathic effect (CPE) was observed microscopically at a ×40 magnification. Virus titers were expressed as TCID_50_ per gram of tissue and then converted to PFU per milliliter by multiplying the TCID_50_ per milliliter by 0.7 (53). Additionally, the viral load was also quantified using real-time PCR with the SYBR green master mix from Promega and SARS-CoV-2 nucleocapsid gene-specific primer pairs and presented as “genome copy numbers” (54). This was done to both corroborate plaque assay values and offer another measure when plaque assays were not sensitive enough.

### Real-time PCR analysis of cytokine transcripts.

RNA was extracted from lungs or spleens collected at sacrifice using the Zymo Direct-Zol RNA miniprep kit (Zymo Research, CA, USA) according to the manufacturer’s instructions. cDNA was synthesized from RNA using the Invitrogen SuperScript III first-strand synthesis system. Synthesis was performed with 0.5 μL (50 ng/μL) of random hexamers, 0.5 μL of 10 mM deoxynucleotide triphosphate, and 4 μL of RNA diluted in a total volume of 15 μL of nuclease-free water. This mix was heated at 65°C for 5 min and chilled on ice, and 1 μL of 10× RT buffer, 1 μL of 0.1 M dithiothreitol, 2 μL of 25 mM MgCl_2_, 0.5 μL of RNase-OUT, and 0.5 μL of SuperScript III enzyme were then added for a final net volume of 10 μL. The cDNA reaction proceeded as follows: 25°C for 5 min, 50°C for 60 min, and 70°C for 15 min. For cytokine levels, specific primer pairs were used for the following cytokines: IFN-γ, TNF-α, IL-13, IL-14, IL-22, IL-9, IL-10, and IL-17. PCR was performed using the StepOnePlus real-time PCR system (Applied Biosystems, Foster City, CA) under the following conditions: 1 cycle at 95°C for 2 min and 40 cycles at 95°C for 3 s and 60°C for 30 s. Each reaction mixture contained 1 μL of diluted cDNA, 10 μL of GoTaq quantitative PCR (qPCR) master mix (Promega), 2 μL of forward and reverse primers, and 7 μL of nuclease-free water. Serial 10-fold dilutions of cDNA were used to establish standard curves for the primers, and temperature melt curve analysis verified the specificity.

### Histopathology and immunohistochemistry.

For histopathology, samples of the lung, liver, and spleen were sectioned and stained with hematoxylin and eosin (H&E). Tissue samples taken from mice infected with M. tuberculosis were also stained with Ziehl-Neelsen stain for the detection of individual bacilli, as done previously (29). For immunostaining, after dewaxing and dehydration, epitope retrieval was performed by heating in a microwave set on 50% power (550 W) for 20 min in a bath of Tris-EDTA buffer (10 mM Tris base, 1 mM EDTA solution, 0.05% Tween 20 [pH 8.4]), after which sections were left to cool in the same buffer and then rinsed with 100 μL of Tris-buffered saline–Tween 20 (TBSTw) (1.5 M NaCl, 0.2 M Tris-HCl, 1% Tween 20). Subsequently, blocking buffer (10 mL of 10% Roche reagent, 5 mL of donkey serum, 1 g of bovine serum albumin [BSA] fraction V, 85 mL of TBSTw) was applied, and sections were left with shaking at 25°C overnight to prevent nonspecific binding. The primary antibody for M. tuberculosis (rabbit anti-Mycobacterium tuberculosis antibody, catalog number ab905; Abcam) was diluted 1:200 in blocking buffer, and 100 μL was applied to each slide. The slides were then incubated with shaking overnight at 4°C in a light-protected container. After incubation, the slides were washed with TBSTw. The secondary antibody (donkey anti-rabbit IgG alkaline phosphatase, catalog number ab7084; Abcam) was diluted in blocking buffer (1:200), and 100 μL of diluted secondary antibody was applied to each slide for 60 min at 25°C with gentle agitation in a light-protected box. The slides were then washed again with TBSTw prior to the use of the Fast Red substrate kit (catalog number ab64254; Abcam), which was used according to the manufacturer’s instructions. After chromogenic detection, the slides were rinsed gently using Dawn dish soap, counterstained with Harris hematoxylin (diluted 1:5) for 2.5 min, and then mounted for analysis.

### Ethics statement.

The animals used in this study were cared for in accordance with the established guidelines and approval of the Institutional Animal Care and Use Committee (IACUC) of the University of Wisconsin—Madison.

### Statistical analysis.

Statistical analysis was done with GraphPad Prism (GraphPad, La Jolla, CA). Data sets that were normally distributed with 3 or more groups were analyzed by one-way analysis of variance (ANOVA) with multiple comparisons to determine *P* values. Normal distributions for cytokine data, viral loads, and bacterial burdens were tested using Anderson-Darling, D’Agostino-Pearson, Shapiro-Wilk, and Kolmogorov-Smirnov tests. Those that were not normally distributed were analyzed using the Kruskal-Wallis test to determine *P* values. *P* values of <0.05 (*), <0.01 (**), <0.001 (***), and <0.0001 (****) were considered significant. Mortality differences were compared using the Kaplan-Meier test. Bacterial burdens were analyzed using Mann-Whitney Student’s *t* test with the *P* value annotations.
