# Supplementary material for: Superinfection with SARS-CoV-2 Has Deleterious Effects on Mycobacterium bovis BCG Immunity and Promotes Dissemination of Mycobacterium tuberculosis
Source: Microbiol Spectr. 2022 Oct 6;10(5):e03075-22. doi: 10.1128/spectrum.03075-22 (PMC9603897; doi:10.1128/spectrum.03075-22)
Supplement: Supplemental file 1 — Fig. S1, Table S1, and Fig. S2. Download spectrum.03075-22-s0001.pdf, PDF file, 0.6 MB [file spectrum.03075-22-s0001.pdf]

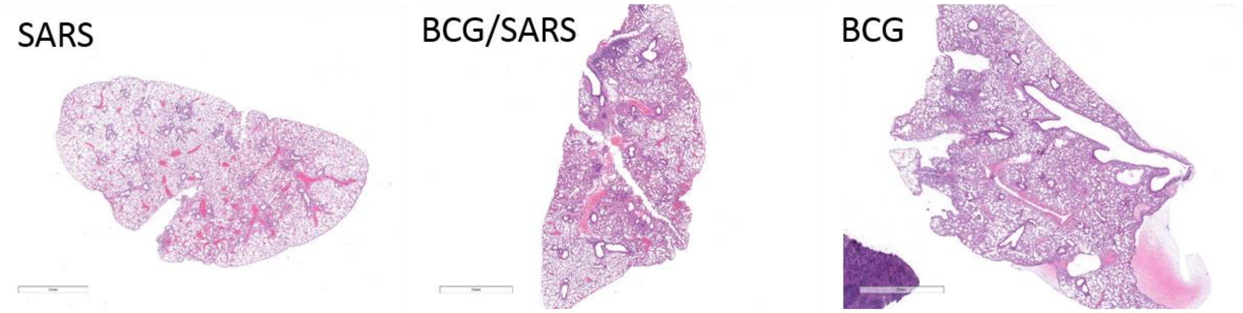

Supplementary figure 1: Hematoxylin and eosin (H&E) stained lung sections from SARS-CoV-2 infected (SARS), BCG vaccinated and SARS-CoV-2 infected (BCG/SARS), and BCG vaccinated alone (BCG) mice sacrificed at time of death between 5 and 7 DPI SARS-CoV-2 infection. All tissues shown are the 8WPV timepoint.

Supplemental Table 1			
group	timepoint	% Lung affected	average
**TB/PBS**	4WPC	20	47
		50	
		60	
		30	
		75	
**TB/SARS-CoV-2**	4WPC	20	23
		15	
		20	
		20	
		40	
**TB/PBS**	8WPC	65	77
		70	
		80	
		80	
		90	
**TB/SARS-CoV-2**	8WPC	70	65
		55	
		65	
		80	
		55	

Supplementary Table 1: Percent affected lung tissue in TB alone and superinfected mice. Hematoxylin and eosin (H&E) stained lung sections from *M. tuberculosis* (TB) and *M. tuberculosis* and SARS-CoV-2 (TB/SARS) infected mice sacrificed at time of death between 5 and 7 DPI SARS-CoV-2 infection. Percent of lung affected by histiocytic inflammation was determined by a trained pathologist for each lung section.

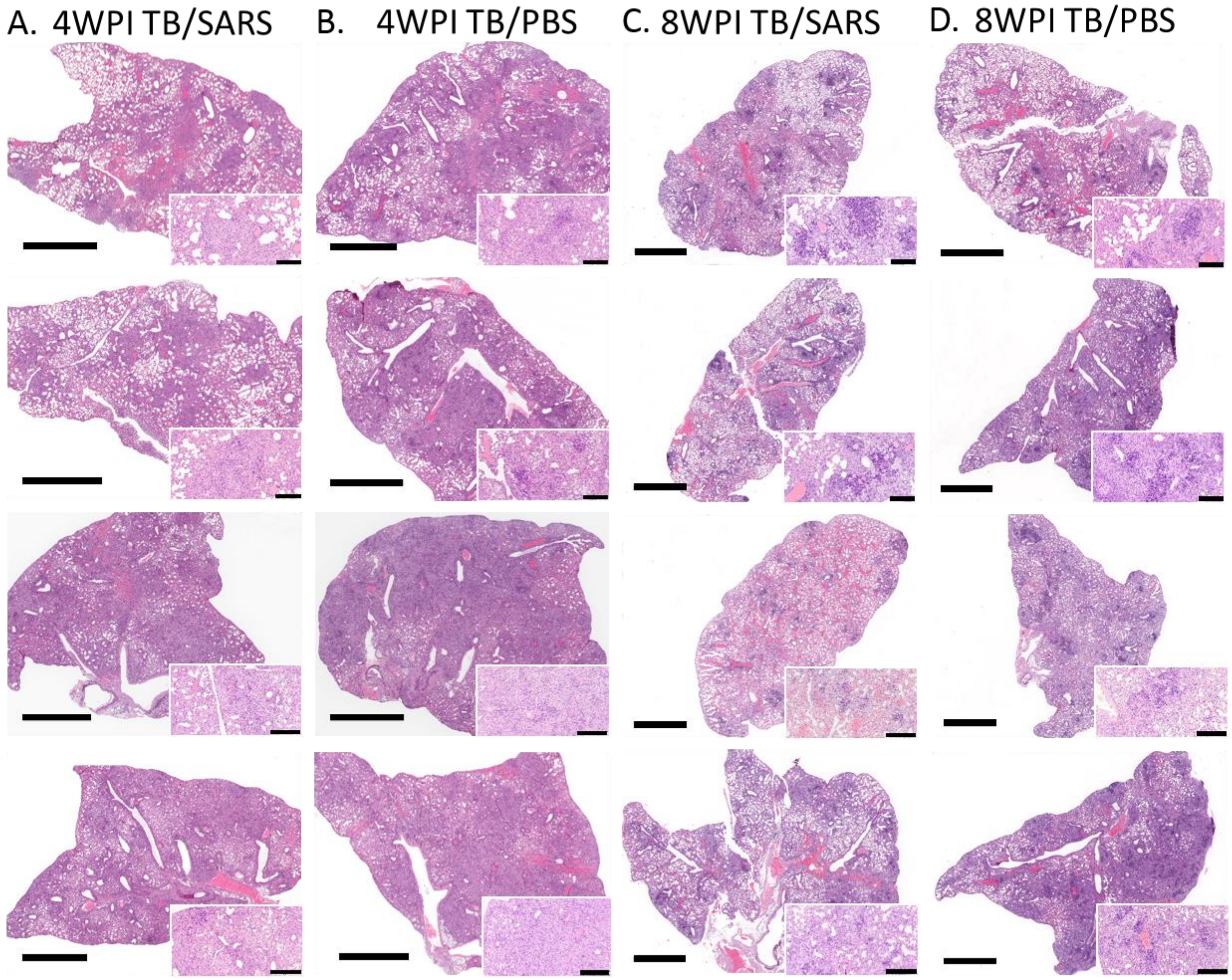

Supplementary Figure 2: Hematoxylin and eosin (H&E) stained lung sections from *M. tuberculosis* (TB) and *M. tuberculosis* and SARS-CoV-2 (TB/SARS) infected mice sacrificed at time of death between 5 and 7 DPI SARS-CoV-2 infection. All scale bars for full-lung image are 2mm. Inserts are either 20x (top two rows with scale bar of 200µm) or 10x (two bottom rows, scale bar of 500µm) to show the tissue features such as organization of inflammatory foci. A) TB/SARS-CoV-2 lung at 4 WPI. B) TB alone lung at 4 WPI C) TB/SARS-CoV-2 lung at 8 WPI. D) TB alone lung at 8 WPI.
